# Women’s awareness of perinatal mental health conditions and the acceptability of being asked about mental health in two regions in India: a qualitative study

**DOI:** 10.1186/s12888-023-05323-5

**Published:** 2023-11-13

**Authors:** Gracia Fellmeth, Pankaj Kanwar, Diksha Sharma, Komal Chawla, Neha DasGupta, Shreyash Chhajed, Chandrakant, Emily C Jose, Anita Thakur, Vikesh Gupta, Omesh Kumar Bharti, Sukhjit Singh, Geetha Desai, Harish Thippeswamy, Jennifer J Kurinczuk, Prabha Chandra, Manisha Nair, Ashok Verma, M Thomas Kishore, Fiona Alderdice

**Affiliations:** 1 National Perinatal Epidemiology Unit, Nuffield Department of Population Health, University of Oxford, Oxford, UK; 2 Department of Psychiatry, Dr Rajendra Prasad Government Medical College, Kangra, Himachal Pradesh India; 3 Department of Obstetrics and Gynaecology, Dr Rajendra Prasad Government Medical College, Kangra, Himachal Pradesh India; 4 British University of Bahrain, Saar, Bahrain; 5 Department of Clinical Psychology, National Institute of Mental Health and Neuro Sciences, Bengaluru, India; 6 State Institute of Health and Family Welfare, Department of Health and Family Welfare, Government of Himachal Pradesh, Shimla, India; 7 Department of Psychiatry, National Institute of Mental Health and Neuro Sciences, Bengaluru, India; 8 School of Nursing and Midwifery, Queen’s University Belfast, Belfast, Northern Ireland

**Keywords:** Acceptability, Awareness, India, Low- and middle-income country (LMIC), Mental health, Perinatal, Pregnancy, Post-partum, Qualitative

## Abstract

**Background:**

Mental health conditions are common during pregnancy and the first year after childbirth. Early detection allows timely support and treatment to be offered, but identifying perinatal mental health conditions may be challenging due to stigma and under-recognition of symptoms. Asking about symptoms of mental health conditions during routine antenatal and postnatal appointments can help to identify women at risk. This study explores women’s awareness of perinatal mental health conditions, their views on the acceptability of being asked about mental health and any preference for specific assessment tools in two regions in India.

**Methods:**

Focus group discussions (FGDs) were conducted with pregnant, post-partum and non-perinatal women in Kangra, Himachal Pradesh (northern India) and Bengaluru, Karnataka (southern India). Settings included a hospital antenatal clinic and obstetric ward, Anganwadi Centres and Primary Health Centres. FGDs were facilitated, audio-recorded and transcribed. Narratives were coded for emerging themes and analysed using thematic analysis.

**Results:**

Seven FGDs including 36 participants were conducted. Emerging themes were: manifestations of and contributors to mental health conditions; challenges in talking about mental health; and the acceptability of being asked about mental health. Difficult familial relationships, prioritising the needs of others and pressure to have a male infant were cited as key stressors. Being asked about mental health was generally reported to be acceptable, though some women felt uncomfortable with questions about suicidality. No preference for any specific assessment tool was reported.

**Conclusions:**

Women face many stressors during the perinatal period including difficult familial relationships and societal pressure to bear a male infant. Being asked about mental health was generally considered to be acceptable, but questions relating to suicidality may be challenging in a community setting, requiring sensitivity by the interviewer. Future studies should assess the acceptability of mental health assessments in ‘real world’ antenatal and postnatal clinics and explore ways of overcoming the associated challenges in resource-constrained settings.

**Supplementary Information:**

The online version contains supplementary material available at 10.1186/s12888-023-05323-5.

## Background

Mental health conditions are the most common morbidities of pregnancy and the first year after childbirth [1]. Prevalence varies across settings but is generally highest in low- and middle-income countries (LMIC) where exposure to social adversity and other risk factors is higher [2]. Mental health conditions during the perinatal period are associated with significant suffering, distress and adverse health outcomes for women and their infants [1, 3]. Early detection can enable timely support and treatment to be offered, which in turn can limit progression of the condition and prevent adverse outcomes for women and their families [4].

Identifying perinatal mental health conditions is challenging, however. Persisting stigma means women may find it difficult to disclose symptoms, while poor awareness around what constitutes a mental health condition may lead to under-recognition of symptoms [5, 6]. Under-detection is of particular concern in LMICs where not only the burden is greater but the association with adverse outcomes is stronger [3].

In some countries including the UK and the USA, women are asked about symptoms of mental health conditions using standardised assessment tools during routine antenatal and postnatal appointments [7, 8]. Systematically assessing for symptoms in this way can help to identify women who might be experiencing a mental health condition and could benefit from support. When considering whether such an approach would work across different cultural contexts, there are several important considerations. Existing assessment tools may need translating and cultural adaptation [9]. Translating complex self-report instruments and ensuring they are easily understandable for diverse groups of women in the target population including those with limited literacy can be challenging [10]. The acceptability of asking about mental health should also be explored as uptake, clinical relevance and the validity of responses to mental health assessments are likely to be compromised if acceptability is low [11].

In India, an estimated one in five pregnant women experiences a mental health condition and 7.6% of women experience suicidal ideation or suicidal behaviours in pregnancy [12, 13]. The need to identify and treat perinatal mental health conditions has become increasingly urgent in light of the Covid-19 pandemic which exacerbated mental health problems globally [14]. Little evidence is available on the validity of screening tools for perinatal mental health disorders in India or on the acceptability of being asked about mental health [15]. One study of perinatal women in Delhi and Maharashtra reported low levels of awareness of perinatal depression, but among those who knew about perinatal depression, the majority agreed that women should be screened during the perinatal period [16]. The current qualitative study builds on these findings by exploring the views of women in two low-income settings in India. The study aims were to: explore women’s awareness of perinatal mental health conditions; understand the perceived acceptability of being asked about mental health; and elicit views on different tools to assess mental health.

## Methods

This was a qualitative study consisting of focus group discussions (FGDs) conducted in two diverse regions of India. The study represents the first phase of the Perinatal Mental Health Study (PMHS), which uses the Maternal and Perinatal Health Research Collaboration India (MaatHRI), a network of sixteen partner hospitals across six states in India [17, 18]. The study was carried out in two regions of India through two collaborating institutions: the Dr Rajendra Prasad Government Medical College (DRPGMC) in Kangra, Himachal Pradesh (HP), northern India, which serves a low-income, rural population; and the National Institute of Mental Health and Neuro Sciences (NIMHANS) in Bengaluru, Karnataka, southern India, which serves a low-income, urban population. FGDs were conducted in May 2022.

### Participants and sampling

FGDs were conducted with women at each study site. Women were eligible to take part if they were aged 18–45 years, could speak the study languages (Hindi in HP; Kannada in Karnataka) and were either pregnant (at any stage of pregnancy), post-partum (had given birth within the past 12 months) or non-perinatal (not currently pregnant and had not given birth within the past 12 months). The reason for including non-perinatal women was to understand views from a wide range of women, not only those who were currently pregnant or had recently given birth. Recruitment was through convenience sampling.

In HP, FGDs were conducted at the DRPGMC hospital antenatal clinic, the DRPGMC obstetric ward and at two Anganwadi Centres in villages approximately 30 km outside of Kangra. In the antenatal clinic, women attending a routine appointment on the day of the FGDs who met the eligibility criteria were invited to participate. On the obstetric ward, eligible women who were present on the ward on the day of the FGDs were invited to participate. At Anganwadi centres, eligible women were invited several days in advance by Anganwadi workers to attend the centre on the scheduled day if they were willing to take part.

In Bengaluru, Karnataka, FGDs were conducted at the NIMHANS Centre for Wellbeing and at two Primary Health Centres. At the NIMHANS Centre for Wellbeing, women receiving care at the centre who met the eligibility criteria were invited a few days in advance to attend on the scheduled day. At Primary Health Centres, eligible women who were attending immunisation clinics on specific days were approached and invited to take part in FGDs.

### Focus group discussions

Eligible women were provided with information about the study including the aims of the research and format of the FGDs. Those who agreed to participate provided written informed consent. The researchers did not know any of the participants prior to the study. A question guide was used as a prompt for discussions, allowing for flexibility in the topics covered (Supplementary File 1). Discussions began with open questions about women’s awareness of perinatal mental health conditions and factors that might contribute to mental health conditions. Questions then moved on to the acceptability of being asked about mental health conditions, followed by specific questions about eight pre-selected tools used to assess for symptoms of mental health conditions. Paper copies of the assessment tools in the study languages were provided. Participants read through the assessment tools themselves or if they preferred, facilitators read them aloud to the group. Participants were asked for their views on the acceptability of the different tools and any specific preferences.

In HP, FGDs were co-facilitated by an English-speaking, female public health researcher (GF); a female research nurse fluent in Hindi, the local dialect of Pahari and English (DS); and a female obstetrician fluent in Hindi and English. FGDs were conducted in private spaces in the antenatal clinic, on the obstetric ward or within the Anganwadi centres. Questions were asked in English and repeated in Hindi, or at times asked directly in Hindi. Discussion between participants was mostly in Hindi and Pahari, with a small number of participants choosing to respond in English. Any comments made in English were repeated in Hindi by the faciliator to ensure everyone could understand and follow the discussion.

In Karnataka, FGDs were facilitated by a female public health researcher (GF), a male clinical psychologist (MTK), a female clinical psychology PhD candidate (not a study author), a female senior staff nurse (not a study author) and a female psychiatric social worker (not a study author). At the NIMHANS Centre for Wellbeing, FGDs were conducted in a private room within the clinic. At the Primary Health Centres, due to space constraints, FGDs were conducted in a corner of the waiting room. Because many of the participants recruited at the Primary Health Centres had limited time available, it was at times necessary to conduct discussions in small groups of two or three, rather than as a larger group. Questions were asked in English and interpreted into Kannada or asked directly in Kannada. Discussions were in Kannada with a small number of participants speaking in English.

FGDs were audio-recorded using a digital voice recorder. One researcher (GF) made notes during and immediately after each FGD on the dynamics of the group, interactions between participants and any non-verbal communication observed.

### Selection and translation of mental health assessment tools

Eight mental health assessment tools were shown to FGD participants: the Kessler Psychological Distress Scale (K10), a measure of generalised psychological distress; the Edinburgh Postnatal Depression Scale (EPDS) and Patient Health Questionnaire (PHQ-9), measures of depression; the Generalised Anxiety Disorder Scale (GAD-7) and Perinatal Anxiety Screening Scale (PASS), measures of anxiety; the PTSD Checklist (PCL-5), a measure of post-traumatic stress disorder (PTSD); the Scale for Assessment of Somatic Symptoms (SASS), a measure of somatic symptoms; and the Suicide Behaviour Questionnaire Revised (SBQ-R), a measure of suicidality. These assessment tools were selected on the basis of a systematic review of validated screening measures in India and through discussion within the research team who have extensive experience of working in the field of mental health in the study settings [15]. Existing Hindi and Kannada language versions of the K10, PHQ-9, EPDS and GAD-7 were adapted to ensure terminology was appropriate to the study settings. The PASS, PCL-5, SASS and SBQ were translated from English into Hindi and Kannada by the study team following World Health Organization (WHO) guidelines for the translation of instruments [19].

### Analysis

Audio recordings were transcribed directly into English by two members of the study team (GF and DS) and an experienced translator (not a study author). Transcripts were imported into *NVivo 11* for coding [20]. Data analysis occurred after all data collection was complete. Data saturation was not formally assessed during the data collection period as the FGDs were conducted over a short period of time. Thematic analysis of the data was conducted following Braun and Clarke’s six consecutive phases [21]. Familiarisation with the data was achieved through reading and re-reading of the transcripts. Any notable features emerging from the data were coded into potential themes and subthemes, and these were then reviewed and refined. Coding was conducted independently by three study team members (GF and PK for transcripts from HP; GF and MTK for transcripts from Karnataka). Particular attention was given to conflicting themes to ensure that voices which were in the minority were reflected in the analysis. Codes generated by the three researchers were compared. Discrepancies in the themes identified were highlighted and consensus was reached among the three researchers by referring back to original transcripts. A final list of themes emerging from both study sites was agreed and shared with the wider research team.

We acknowledge the possibility of unconscious biases held by the research team influencing data collection and interpretation. For each FGD, at least one facilitator came from the local community, ensuring cultural sensitivity. Of the three researchers conducting data analysis, two have extensive experience of working in mental health in the respective local settings and carrying out qualitative studies (PK and MTK) and the third is experienced in conducting qualitative studies across diverse low-income settings globally (GF). Results are reported according to the Consolidated Criteria for Reporting Qualitative Research (COREQ) checklist (Supplementary File 2) [22].

### Findings

Seven FGDs were conducted including a total of 36 participants (14 pregnant, 15 post-partum and 7 non-perinatal) (Table 1). Basic demographic information was obtained for 29 participants. The age range among those who provided demographic information was 24–40 years and educational background ranged from completion of primary to post-graduate degrees. Five participants lived in nuclear households and the remaining 24 lived in joint households with extended families, most commonly with parents-in-law. FGDs lasted between 30 min and one hour.

**Table 1:** Summary of focus group discussions conducted

FGD	Location	Setting	Participants
1	Village near Kangra, HP	Anganwadi Centre (Community; Rural)	4 pregnant women 5 post-partum women 3 non-perinatal women
2	Village near Kangra, HP	Anganwadi Centre (Community; Rural)	1 pregnant woman 3 post-partum women 1 non-perinatal woman
3	Kangra, HP	Antenatal clinic (Hospital; Rural)	6 pregnant women
4	Kangra, HP	Obstetric ward (Hospital; Rural)	1 pregnant woman 2 post-partum women
5	Bengaluru, Karnataka	NIMHANS Wellbeing Centre (Community; Urban)	3 non-perinatal women
6	Bengaluru, Karnataka	Primary Health Centre (Community; Urban)	2 pregnant women 1 postpartum woman
7	Bengaluru, Karnataka	Primary Health Centre (Community; Urban)	4 postpartum women

Three over-arching themes emerged from participants’ narratives: manifestations of perinatal mental health conditions and their contributing factors; challenges of talking about mental health; and the acceptability of being asked about mental health. These three themes and the sub-themes identified within each are described below.

### Theme 1: Manifestations of perinatal mental health conditions and contributing factors

Although some participants said they knew little about mental illness, generally women were aware that mental disorders can occur during the perinatal period:

*I heard many things about mental issues ladies are having during pregnancy. Even I also have mental health issues during pregnancy.* [Post-partum woman; Karnataka]

Participants described a range of ways in which mental health conditions may manifest including wanting to be alone, being withdrawn and a lack of engagement.

*Sometimes…they feel very, very low. They don’t like to stay there [home], they want to go away from home. They want to stay alone.* [Post-partum woman; HP]

*She thinks that everything in this world is of no use and she wants only peace… If she doesn’t get peace in her family, then she goes to the other [place], where she can be better. To that place where she can get calm and peace, and…isolate.* [Non-perinatal woman; HP]

Loss of pleasure or interest was another common feature.

*When signs of depression appear in a person they become short-tempered, they don’t want to think, the things that excited them now no longer excites them.* [Pregnant woman; HP]

*A child may smile at you, but you may not receive that smile.* [Non-perinatal woman; Karnataka]

Over-thinking was mentioned, alongside feelings of stress, tension and fatigue.

*Mental health is the tension and stress that we face. We do face a lot of tension at home. We will not know how to handle it.* [Non-perinatal woman; Karnataka]

*Body pain, brain fatigue, feeling tired – without working feeling tired, thinking negative a lot, a lot of thinking.* [Unrecorded perinatal status; HP]

Participants identified a wide range of factors contributing to mental health conditions. Women commonly described the challenges of adjusting to a new family and difficult relationships with in-laws upon moving into their husbands’ family home after marriage or childbirth.

*After marriage, the first depression is related to mother-in-law. This is the reality of the life. Whether mother-in-law is good or bad, just thoughts in our mind related to her. You have to adjust in that family.* [Non-perinatal woman; HP]

Most women felt insufficiently supported by their extended families. Women emphasised the importance of remaining calm and positive during pregnancy to ensure their own and the baby’s health.

*Whatever issues we might be having, we should keep them aside and stay happy for the baby’s health… [The family] should not pressurize us, they should not fight with us. They should keep us happy.* [Post-partum woman; Karnataka]

*During pregnancy it’s a high risk [time], actually. Getting support from our family and husband is very…is an important thing in the pregnancy.* [Unrecorded perinatal status; Karnataka]

Many women described being expected to tend to the needs of everyone else first, and having to put their own needs last.

*The woman has to listen to everyone. Husbands, family, everyone first and she comes last.* [Pregnant woman; HP]

*The rest of the family members have to be put on the, like, priority list. After that the husband comes next. We need to respect what other family members say, and after that we have to listen to what our husband asks us.* [Pregnant woman; HP]

Along with a lack of support, the expectations placed on women led to tension and anxiety.

*When everyone is expecting from you then you feel pressurized… It can happen to anybody. When everyone around is expecting from only one person, then that person will feel pressurised, maybe.* [Unrecorded perinatal status; HP]

*She is having 1 year old child and she has to do household work and side-by-side dealing with her husband who is having short temper and also some family issues, all leading to anxiety.* [Post-partum woman; HP]

Several women spoke about the societal pressure to have a male child and how this could cause distress.

*When there is a boy, they celebrate so much and if there is a girl, they have so much sorrow.* [Pregnant woman; HP]

*[The pressure to have a boy] is 90% from family, 10% from the woman. She will be mentally tortured.* [Unrecorded perinatal status; HP]

*The mother accepts any baby. But it’s the other extended family members. They believe that…their family progresses provided they have a male child, and not the female child.* [Non-perinatal woman; Karnataka]

### Theme 2: Challenges of talking about mental health

There was general agreement that talking openly could help those suffering from a mental health condition. Women alluded to conversations with close friends and family rather than with healthcare professionals.

*We have to talk about such type of topics. Human beings are opened by speaking.* [Post-partum woman; HP]

*We feel light only if we share [our issues].* [Post-partum woman; Karnataka]

*Even if I don’t have any solution to the problem, you might have the solution to that problem. …When a person starts speaking it also gives you confidence to express your anxiety.* [Post-partum woman; HP]

Women felt that there should be no shame in talking about mental health conditions.

*If you are not talking, then that is why the problem is prevailing at this time. If you are not talking about it then definitely this is going to lead to depression in the society.* [Pregnant woman; HP]

*Why feel shame about it? It’s nothing to be ashamed.* [Post-partum woman; HP]

Yet despite the perceived benefits, women recognised the significant challenges of talking about mental health. Many participants considered mental health a private matter to be kept within the family.

*They don’t tell anyone. They just keep it within their family. They will not share it outside the house. Within the house they will just solve that problem, they will not include another person.* [Non-perinatal woman; HP]

A key reason for not disclosing symptoms was the fear of being mocked by others.

*A person wants to speak his heart, but our society is such that it will call him a lunatic. Everyone makes fun of him. They just become a laughter stock, the people who talk about such things.* [Pregnant woman; HP]

*[A person with depression] will think, when I talk with anyone else about my problem, then the other person will not understand my suffering, they will say that you are stupid, mad…* [Post-partum woman; HP].

### Theme 3: Acceptability of being asked about mental health

Generally, women felt that being asked about symptoms of mental health conditions during the antenatal or postnatal period was a good idea.

*If somebody’s asking about mental health, we also feel good, we will also feel happy that somebody’s asking about our health.* [Post-partum woman; Karnataka]

*Definitely it will be a good thing if someone comes for antenatal check-up and we get the screening for depression, so that she doesn’t advance to disease of depression* [Pregnant woman; HP].

It was suggested that informing women in advance could increase the acceptability of being asked. Women felt that assessments should form part of the routine health check. Some suggested including mental health questions at every appointment, but others were more ambivalent and a few felt that questions could cause discomfort or even distress.

*Women will not answer these questions…because of the shame* [Perinatal status unrecorded; HP].

*It would be difficult to ask. They are not able to speak openly about it.* [Non-perinatal woman; HP]

There was some concern that women might feel obliged to answer questions even if they did not want to. Familiarity with the person asking the questions was considered important in putting individuals at ease.

*We need to have that comfortable relationship with the person first. Because when we are comfortable with the person, only then the other person can express themselves to the other person.* [Pregnant woman; HP]

One participant stressed the importance of asking women individually rather than in a group.

*In a group they will not share their feelings, but if we ask them individually they might share their experience or their feelings.* [Non-perinatal woman; HP]

Another participant suggested that questions about risk factors for mental health conditions, such as the pressure to have a boy and relationship challenges, should also be asked.

*You should also include another thing…regarding the [cultural preference] for a boy child and family relations* [Non-perinatal woman; HP].

### Preference for tools

In general, women reported that the mental health assessment tools were clear and feasible to answer. Overall, there was no clear preference for any specific tool.

One participant spoke positively of the K10 questions:

*Because they are in a single sentence, we can respond easily.* [Non-perinatal woman; Karnataka]

For depression, some preferred the EPDS while others preferred the PHQ-9. Most participants had no preference for either tool.

*This one [EPDS] because it includes if a person is suffering from nervousness, any trouble.* [Perinatal status unrecorded; HP]

*PHQ is easy because most of the questions are related to my case.* [Post-partum woman; Karnataka]

One question on the PHQ-9 which asks about feeling “that you are a failure or have let yourself or your family down” was felt to be potentially upsetting.

*If a pregnant lady is asked the question saying I am not worthy, what if she develops depression?* [Non-perinatal woman; Karnataka]

Both anxiety measures seemed to be acceptable. Slightly more participants expressed a preference for the GAD-7, although there were no negative comments about the PASS.

*I like this one [GAD-7] because it’s short and simple. It covers each and every aspect in a very short span. Also it doesn’t count the number of days.* [Perinatal status unrecorded; HP]

*This [PASS] questionnaire is easy because it’s based on the child-related [issues], you know? About the future of the child… I’m already dealing with anxiety and these questions are related to my situation, so I’m able to answer them all.* [Perinatal status unrecorded; HP]

The PCL-5 and the SASS were considered acceptable and clear, but questions about self-harm and suicidality were more divisive. The SBQ in particular was felt to be unacceptable by a number of women, as it asks in detail about suicidal thoughts and intent. Some women felt these questions could cause distress and should not be asked.

*Many people think that during the time when you’re antenatal you should not talk about suicidal thoughts… It has detrimental effects on the baby also, to talk about it.* [Pregnant woman; HP]

*Pregnant women should not be asked such questions. It will cause more tension. The reason is, that they might get upset when you talk about death.* [Non-perinatal woman; Karnataka]

However, others felt it was acceptable to ask about suicidal ideation.

*It’s not a problem to answer this. For everyone, no. But for me, I’m ok to answer this.* [Unrecorded perinatal status; HP]

*For all the questions, they’re ok to ask during pregnancy, including the suicidal thoughts or depression things. They are able to answer it if you ask those questions.* [Post-partum woman; Karnataka]

One participant suggested self-completion of mental health assessments would be preferable over verbal administration by a healthcare professional.

*Sometimes we are not able to speak out our feelings but we are able to write what we think, our inner feelings* [Pregnant woman; HP].

### Observations during FGDs and analysis

FGD questions were deliberately phrased indirectly (for example, “How would you know if someone had a mental health condition?”) to avoid participants feeling pressured to share personal experiences. Analysis of the transcripts showed that when discussing symptoms, women mostly used a third person narrative (for example, “*They want to stay alone*.”). By contrast, when discussing contributing factors, women provided more personalised perspectives, often sharing examples from their own lives (for example, “*My mother-in-law troubles me…*”). Dynamics between participants varied across FGD groups but often one or two women contributed a lot while others spoke less. Efforts were made to encourage all women to join in the discussion, but nevertheless some women remained more reserved.

## Discussion

This qualitative study provides insights into women’s perceptions of perinatal mental health conditions, the contributing factors and the acceptability of being asked about mental health in two settings in northern and southern India.

Overall, participants were aware of mental health conditions during the perinatal period and described a variety of behavioural, affective and cognitive symptoms. Compared to other Indian studies, knowledge around mental health conditions was high in our group. For example, in a study of perinatal women in Delhi, Maharasthra, Mangalore and Karnataka, most participants considered depression to be a normal part of the perinatal period that did not warrant treatment [23]. In another study from Delhi and Maharashtra, less than 10% of participants were aware of perinatal depression, with lowest awareness among those with lower incomes and lower educational levels [16]. It is also possible that higher levels of awareness in our study were observed due to our participants being less representative of the general population. For example, participants recruited through Anganwadi Centres in HP were a self-selecting group who chose to take part in FGDs and may have had a particular interest in mental health, while those recruited through the NIMHANS Wellbeing Centre may have had greater knowledge of mental health conditions by virtue of having attended the Centre. Several participants also had high educational backgrounds including some with post-graduate qualifications, which may further explain the high level of awareness.

Women described a number of factors contributing to mental health. A perceived lack of support from partners and in-laws and the expectation to prioritise the needs of other family members were common experiences. The pressure to bear a son rather than a daughter was a further stressor for many. While many women said they personally had no gender preference, they often described parents-in-law, partners and the wider family being disappointed upon the birth of a girl. These socio-cultural factors have been identified as risk factors for perinatal mental health conditions across South Asian settings [12, 24, 25]. Malhotra and Shah describe the social disadvantage women in India face, including “pressures…created by their multiple roles and the unremitting responsibility of caring for others” [26]. These experiences may lead to a sense of disempowerment with a resultant loss of agency, further impacting upon mental health [27].

While women recognised the benefits of talking openly about mental health, they also described significant challenges. Participants described stigmatising and derogatory attitudes, reporting that individuals with mental health conditions are labelled as ‘lunatic’. Many of these themes were highlighted in a previous qualitative study from Bengaluru, where fear of being labelled ‘mad’ was a barrier to perinatal women disclosing mental health issues [28]. Stigma may also have influenced our FGDs: although many participants appeared at ease speaking about mental health, others may have felt less comfortable discussing this topic within a group. Self-perceived stigma around perinatal mental health conditions has been identified elsewhere in India as a key barrier to help-seeking [23]. Our transcripts showed a shift in narratives from a third-person perspective when discussing symptoms to a first-person perspective when discussing contributing factors, suggesting women may have found it easier to talk about everyday stressors than to discuss mental health symptoms. This is relevant in light of one participant’s suggestion that risk factors for mental health conditions should be assessed alongside symptoms. Enquiring about issues such as familial conflict and social support could potentially offer a gentler window into a broader conversation about mental health.

Despite stigma, being asked about mental health was considered acceptable to most women. Such views were also found in a previous study in Bengaluru, in which perinatal women reported they would be pleased if a health professional asked about their mental health, but “nobody asks, so we don’t share” [28]. Participants in our study reported that mental health assessment tools were generally clear and easy to understand, and no overall preferences were evident for any specific tool. However, there are important caveats to our findings. Firstly, social desirability may have led to participants avoiding giving any critical feedback. Secondly, although participants were asked to read through each tool, it was not feasible to discuss each of the eight tools in detail or go through each one question-by-question. Therefore it was not possible to explore in depth whether individual questions were understood or how they were interpreted. Finally, women were not asked to complete the assessment tools themselves, but rather to give general opinions on their acceptability. Women’s views on the various assessment tools may have been different if they had been asked to complete them. These caveats are important because other studies have highlighted the challenges in cross-cultural application of many mental health assessment tools. The EPDS in particular has been found to be challenging due to difficult-to-translate, abstract phrases (for example, “things have been getting to me”) and the complexity of its response options [9, 29–31].

Not all women were comfortable with the idea of being asked about suicidal ideation. Some felt these questions could cause distress during a sensitive time for women. Strongest reactions related to the SBQ-R, but the final item of the PHQ-9 (“thoughts that you would be better off dead, or of hurting yourself”) was also considered unacceptable by some. These concerns were voiced by a minority, and others felt that questions about suicidality were important to ask. Rates of maternal suicide in India are high and identifying women at risk is important [32]. Because suicidality can occur independently of depression, asking about depressive symptoms alone is not always enough to identify women at risk of suicide [33, 34]. Further work is needed to explore the least intrusive and least distressing ways to assess suicidality, and the methods that are most likely to generate a true response in this highly stigmatised issue. Asking about suicidality requires sensitivity, empathy and adequate training, with an awareness that questions may be uncomfortable and even distressing for the interviewee.

### Implications for research and practice

In resource-constrained settings, assessing mental health within clinics where staff are often already over-stretched presents considerable challenges. Stigma and time pressures may impact women’s willingness to discuss their mental health within the context of a clinic appointment. Holding discussions in a private space, informing women in advance and having a supportive, familiar health professional administer the questions may help to put women at ease, though these may not always be feasible. Future research should explore the challenges of implementing routine mental health assessments in resource-constrained community settings.

### Limitations

The study has a number of limitations. It was not possible to separate women into different FGD groups on the basis of age, parity or educational level, and some women – for example those who were older or more experienced as mothers – may have felt more at ease than others at speaking within the group. We used various strategies to encourage participants to contribute to the discussion, including explaining confidentiality and that all views are equally important, using ice-breakers, informal language and humour, and directing some of the questions at the quieter participants. Stigma may also have impacted upon women’s willingness to speak within a group. Individual interviews may be better suited to eliciting in-depth understanding of mental health. FGDs were conducted in primary care settings which are often crowded, noisy and have little privacy. Many women had infants with them, and some were unable to remain for the full FGD duration due to long journeys home, jobs or domestic obligations. These factors may have led to lower engagement with the discussions and less in-depth responses. However, these are the realities of conducting FGDs in many low-resource settings [28], especially when attempting to reach groups who are often marginalised and excluded from research. Social desirability may have influenced the discussions and participants may not have been representative of the wider community. We did not assess the acceptability of asking women to complete about assessments in the ‘real life’ scenario of a busy antenatal or postnatal clinic, where women are likely to have less time. Mistranslation or misunderstanding of data may have occurred as a result of working across different languages, although we conducted checks to ensure interpretations and translations were as accurate as possible. Due to the lack of follow-up of participants, it was not possible to ask participants for comments or feedback on the transcripts or findings.

## Conclusion

This qualitative study explored women’s awareness of mental health conditions and the acceptability of being asked about mental health. Difficult familial relationships, being expected to prioritise the needs of others and pressure to bear a male infant are key stressors for perinatal women in these settings in India. Stigma around mental health remains significant. Being asked about mental health conditions was generally felt to be acceptable, but questions relating to suicidal behaviours may be challenging in a community setting and require sensitivity by the interviewer. No preference for a specific mental health assessment tool was expressed; further research is required to confirm this and explore how questions are interpreted. Future studies should assess the acceptability of conducting mental health assessments in antenatal and postnatal clinics and explore ways of overcoming the associated challenges in resource-constrained community settings.

### Electronic supplementary material

Below is the link to the electronic supplementary material.

Supplementary Material 1 - COREQ Checklist

Supplementary Material 2 - FGD Questions Guide

## Data Availability

The datasets generated and analysed during the current study are not publicly available due to the sensitive nature of the data but are available from the corresponding author on reasonable request.

## Abbreviations

DRPGMC: Dr Rajendra Prasad Government Medical College
EPDS: Edinburgh Postnatal Depression Scale
FGD: Focus group discussion
GAD-7: Generalised Anxiety Disorder Scale
HIC: High-income country
HP: Himachal Pradesh
IEC: Institutional ethics committee
K10: Kessler Psychological Distress Scale
LMIC: Low- and middle-income country
MaatHRI: Maternal and perinatal Health Research collaboration India
NIMHANS: National Institute of Mental Health and Neuro Sciences
PASS: Perinatal Anxiety Screening Scale
PCL: PTSD Checklist
PHQ-9: Patient Health Questionnaire-9
PTSD: Post-traumatic Stress Disorder
PMHS: Perinatal Mental Health Study
SASS: Scale for Assessment of Somatic Symptoms
SBQ: Suicide Behaviour Questionnaire
WHO: World Health Organization
