# Supplementary material for: Women’s awareness of perinatal mental health conditions and the acceptability of being asked about mental health in two regions in India: a qualitative study
Source: BMC Psychiatry. 2023 Nov 13;23:829. doi: 10.1186/s12888-023-05323-5 (PMC10644637; doi:10.1186/s12888-023-05323-5)
Supplement: Supplementary file 2 — Supplementary Material 2 - FGD Questions Guide [file 12888_2023_5323_MOESM2_ESM.pdf]

FGD Questions Guide

1. Welcome

- Welcome participants
- Thank participants for attending
- Facilitators introduce themselves
- Participants introduce themselves

2. Overview / introduction of the topic

- Explain purpose of FGD (to elicit views around mental disorders; gather feedback on screening tools)
- Explain what results will be used for (inform next stages of research; help select tool to identify women with mental disorders; using these to find out how common mental disorders are and who is at risk)

3. Ground rules

- Free to share views; there are no wrong answers; 'negative' comments are just as valid and helpful as 'positive' comments; reminder that participants can decide to leave anytime (with no need to provide reason for leaving); confidentiality (no part of discussion to be shared outside the room).
- Audio-recorder: explain need to record session. No names will be used on our reports therefore assuring complete confidentiality.

4. Questions

<u>Views around mental disorders</u>

- Have you heard of mental health conditions / illnesses of the mind?
- How would you know if someone might be suffering from a mental health condition? What might the symptoms be?
- What do you think might cause mental health conditions? Why do they occur?
- Have you heard of mental health conditions occurring during pregnancy / after childbirth? Do you think women might be more at risk or less at risk during this time (& why)?
- Do you feel it's ok/acceptable to talk about mental health conditions – with family, friends, healthcare workers?
- If someone has a mental health conditions disorder, what might help them? (Prompts if appropriate: where might they go for help; is there anything that can help them to suffer less?)

<u>Views around self-report measures</u>

- Read out self-report measures / or give participants time to read them (according to preference) before asking:
- What are your thoughts about these measures?
- How easy/difficult are they to understand?
- Is there anything confusing, unclear, hard to understand? Or anything you find especially good, easy?
- What do you think about the response categories?
- Do you think the questions fit with what you someone with a mental health condition would be feeling?
- Are there any questions about any symptoms/aspects that are missing?
- How would you feel if you were asked to answer these questions? How do you think women would feel if they were asked to answer these questions when they come to their antenatal/postnatal clinic?
- Is there any measure that you like most / like least (and if so why)?

5. Conclusion

- Summarize discussion; ask if anything has been missed.
- Thank participants for their time.
